# Oxygen Radicals Elicit Paralysis and Collapse of Spinal Cord Neuron Growth Cones upon Exposure to Proinflammatory Cytokines

**DOI:** 10.1155/2014/191767

**Published:** 2014-06-23

**Authors:** Thomas B. Kuhn

**Affiliations:** Department of Chemistry and Biochemistry, University of Alaska Fairbanks, 900 Yukon Drive, Reichardt Building, Room 194, Fairbanks, AK 99775-6150, USA

## Abstract

A persistent inflammatory and oxidative stress is a hallmark of most chronic CNS pathologies (Alzheimer's (ALS)) as well as the aging CNS orchestrated by the proinflammatory cytokines tumor necrosis factor alpha (TNF*α*) and interleukin-1 beta (IL-1*β*). Loss of the integrity and plasticity of neuronal morphology and connectivity comprises an early step in neuronal degeneration and ultimate decline of cognitive function. We examined *in vitro* whether TNF*α* or IL-1*β* impaired morphology and motility of growth cones in spinal cord neuron cultures. TNF*α* and IL-1*β* paralyzed growth cone motility and induced growth cone collapse in a dose-dependent manner reflected by complete attenuation of neurite outgrowth. Scavenging reactive oxygen species (ROS) or inhibiting NADPH oxidase activity rescued loss of neuronal motility and morphology. TNF*α* and IL-1*β* provoked rapid, NOX-mediated generation of ROS in advancing growth cones, which preceded paralysis of motility and collapse of morphology. Increases in ROS intermediates were accompanied by an aberrant, nonproductive reorganization of actin filaments. These findings suggest that NADPH oxidase serves as a pivotal source of oxidative stress in neurons and together with disruption of actin filament reorganization contributes to the progressive degeneration of neuronal morphology in the diseased or aging CNS.

## 1. Introduction

A spreading inflammatory reaction accompanied by oxidative stress is prevalent in most chronic CNS diseases and acute CNS trauma as well as in the aging CNS [1–3]. The proinflammatory cytokines TNF*α* (tumor necrosis factor *α*) and IL-1*β* (interleukin-1*β*) exert pleiotropic functions both in CNS development and CNS pathogenesis [4–6]. Persistent high-level expression of TNF*α* and IL-1*β* is important to the progressive degeneration of neuronal connectivity and loss of neuronal plasticity ultimately leading to cognitive decline. The role of TNF*α* and IL-1*β* as inducers of apoptotic events is well documented, whereas the recognition of their morphogenetic function is more recent [7]. TNF*α* and IL-1*β* released from microglia cells inhibited neurite outgrowth, reduced branching, and caused neurite retraction in cultures of Neuro2A cells or primary hippocampal neurons [8, 9]. The intricate pattern of neuronal connectivity innate to cognitive function rests as much on the integrity and stability of the axonal and dendritic architecture as on the plasticity of motile structures to maintain, form, or regenerate connections, which is intimately linked to the dynamic reorganization of the actin cytoskeleton [10, 11]. ROS intermediates greatly affect both the dynamics and organization of actin filaments during oxidative stress of physiological redox signaling [12, 13]. TNF*α* paralyzed actin filament reorganization in neuroblastoma cells due to oxidative damage, whereas physiological levels of ROS intermediates seem to be necessary for proper growth cone motility [14, 15]. TNF*α* and IL-1*β* potently stimulate NADPH oxidase (NOX) activities in neurons and glia cells often localized in coalescing lipid rafts [16, 17]. The members of the NADPH oxidase (NOX) family (NOX1–5, DUOX1/2), defined by the large membrane flavoprotein gp91^phox^ of the phagocyte NAPDH oxidase (NOX2), are ubiquitously expressed in all cell types and have emerged as principal ROS sources both in cellular signaling and disease progression in response to cytokines, growth factors, and hormones [18, 19]. Functional NOX requires an intricate assembly between two membrane proteins (a NOX isoform, gp22^phox^) and several cytosolic factors (p47^phox^, p40^phox^, p67^phox^) under the regulation of the small GTPases Rac1 or Rac2 [20]. Rho GTPases harbor a dual role in cytokine signaling as regulators of both NOX assembly and the reorganization of actin filament structures [21–23]. In light of these reports, we examined whether ROS intermediates generated by NOX activities in neuronal growth cones are implicated in mediating the neurotoxic effects of TNF*α* or IL-1*β* on neurite outgrowth. A mechanistic understanding of the detrimental consequences of TNF*α* and IL-1*β* on neuronal connectivity in the CNS neurons is vital to intervene with progressive neurodegeneration in the aging or diseased CNS.

## 2. Materials and Methods

### 2.1. Reagents

Unless state otherwise, all reagents were purchased from Sigma (St. Louis, MO). Dulbecco's Modified Eagle Medium (high glucose DMEM), Leibovitz's L-15, and isopropyl *β*-D-thiogalactopyranoside were obtained from Invitrogen Corporation (Carlsbad, CA). Characterized fetal bovine serum (FBS) was from Hyclone (Logan, UT) and it was heat-inactivated according to the manufacturer. Laminin was from Roche Diagnostics Corporation (Indianapolis, IN). The redox sensitive fluorescent indicators carboxymethyl-2′,7′-dihydrodichlorofluorescein diacetate, 2′,7′-dihydrodichlorofluorescein diacetate, and Calcein Blue were obtained from Molecular Probes (Eugene, OR). Human recombinant tumor necrosis factor *α* (TNF*α*) and interleukin-1*β* (IL-1*β*), Diphenylene iodonium (DPI), N-acetyl-L-cysteine (NAC), and Manganese(III) tetrakis(4-benzoic acid) porphyrin chloride (MnTBAP) were purchased from Calbiochem (San Diego, CA). Polyclonal rabbit anti-TNF receptor I, polyclonal rabbit anti-TNF receptor II, polyclonal rabbit anti IL-1 receptor type I, polyclonal goat anti-NOX2, polyclonal goat anti-p47^phox^ including respective blocking peptides were all from St. Cruz Biotechnology (St. Cruz, CA). A monoclonal mouse anti-p44/42 MAPK, a monoclonal mouse anti-phospho p44/42 MAPK, and a monoclonal mouse anti-phospho JNK were from Cell Signaling Technologies (Danvers, MA). Fluorescein or rhodamine-labeled goat anti-rabbit and goat anti-mouse IgG (resp.) were from Chemicon (Temecula, CA), whereas rhodamine-conjugated phalloidin was obtained from Cytoskeleton Inc. (Denver, CO). The polyclonal rabbit anti-p67^phox^ was purchased from Abcam (Cambridge, MA).

### 2.2. Spinal Cord Neuron Cultures

Dissociated, low-density cultures of spinal cord (SC) neurons were established as described [24]. Briefly, spinal cord tissue was dissected from 7 day-old chick embryos (E7) and a single cell suspension obtained after enzymatic digestion (0.5 mg/mL trypsin, 2 mM EDTA, 10 min, 37°C) followed by trituration. After preplating (1 h, 37°C, 5% CO_2_ atmosphere, high glucose DMEM, pH 7.3/10% FBS), nonadherent cells (predominantly SC neurons) were collected (3 min, 200 ×g_max⁡_) and resuspended in SC medium (high glucose DMEM pH 7.3, 10% FBS, 12 nM fluorodeoxyuridine, 30 nM uridine, and 1% N3 nutrient supplement) [25]. SC explants were prepared by pushing freshly dissected E7 spinal cord tissue through a wire-mesh (50–75 *μ*m). Dissociated SC neurons (75,000 cells/mL) or SC explants (2 per culture) were plated onto glass cover slips (22 × 22 mm^2^, number 1, Carolina Biological Supply Company, Burlington, NC) mounted over a 1.5 cm hole drilled into the bottom of a 35 mm culture dish. Glass cover slips were treated with poly-D-lysine (100 *μ*g/mL, borate buffer pH 8.4, 30 min, RT) prior to coating with laminin (5 *μ*g/cm^2^, 30 min, RT). These SC neuron cultures contained less than 5% nonneuronal cells with no immunoreactivity against microglia markers.

### 2.3. Growth Cone Particle Preparations

Preparations of highly enriched growth cone particles were obtained from freshly dissected whole chick embryo brains (E10-12) [26, 27]. After homogenizing (Dounce homogenizer, ice-cold 5 mM HEPES pH 7.3, 1 mM MgCl_2_, 0.32 M Sucrose, 6–8 volumes per wet weight), homogenates were centrifuged (15 min, 1600 ×g_max⁡_, 4°C). Resulting supernatants were overlaid onto 5 mM HEPES pH 7.3, 1 mM MgCl_2_, and 0.75 M sucrose and then centrifuged (150,000 ×g_max⁡_, 1 h, 4°C), and material at the interface was collected. After dilution (6-7 times in low sucrose buffer), the suspension was overlaid onto MaxiDense (4-5 sample volumes per volume Maxidense) and centrifuged at 40,000 ×g_max⁡_ (1 h, 4°C), and growth cone particles (GCPs) were collected on top of the MaxiDense cushion. GCPs were resuspended in Kreb's buffer (145 mM NaCl, 5 mM KCl, 1.2 mM NaH_2_PO_4_  × H_2_O, 1.2 mM MgCl_2_, and 5 mM HEPES pH 7.3) and protein concentration determined (BCA assay, Thermo Scientific, Rockford, IL, USA).

### 2.4. Measurement of Neurite Lengths

Dissociated SC neurons were grown after the onset of neurite outgrowth indicated by the majority of cells extending at least one process longer than 2 cell body diameters. Cultures were incubated (1 h) with pharmacological agents, followed by bath application (6–8 h) of 100 ng/mL TNF*α*, 100 ng/mL IL-1*β*, or 10 *μ*g/mL ovalbumin. After fixation (2% glutaraldehyde), the length of the longest neurite per neuron was measured of at least 50 randomly selected SC neurons only considering processes adhering to the following criteria: (i) emerging from an isolated cell body, (ii) longer than two cell diameters, and (iii) no contact to other neuronal processes or cell bodies. The distribution of neurite length in a population of SC neurons was obtained by plotting the percentage of neurons with neurites longer than a given length against neurite length [28]. As a characteristic for neurite outgrowth under a given condition, the neurite length reached by 50% of neurite-bearing SC neurons (NL_50_) was calculated as criteria for statistical significance [29]. Purified, recombinant Rac1^V12^-GST, Rac1^N17^-GST, or GST were introduced into freshly dissociated SC neurons (7–10 mg/mL, 500,000 cells, 200 *μ*L 50 mM Tris-Cl pH 7.5, 100 mM NaCl, and 5 mM MgCl_2_) by trituration loading at the time of plating [26]. Following trituration, SC neurons were immediately transferred in SC medium and plated as described above. For each condition, measurements were performed in duplicate cultures from two independent dissections.

### 2.5. Measurement of Growth Cone Advance

Dissociated SC neurons (12 to 16 h in culture) were transferred into observation medium (Leibovitz'L15 without phenol red, pH 7.4, 5 mg/mL ovalbumin, 1% N3), overlaid with light mineral oil to avoid evaporation, and placed onto the microscope stage equilibrated at 37 ± 0.2°C (Nikon TE2000 U). After 15-minute recovery, images of advancing growth cones (20x, phase contrast) were acquired at 3-minute time intervals for a 30 min time period (Coolsnap*fx*, Photometrics, Tuscon, AZ). Cytokines (100 ng/mL TNF*α*, 100 ng/mL IL-1*β*) or 10 *μ*g/mL Ovalbumin were bath-applied at *t* = 8 min. The extension of the growth cone/neurite boundary (*μ*m) was measured as a function of time (min) (Metamorph Software, Meridian Instrument Co, Kent, WA). At least 20 growth cones were monitored for each condition in duplicate cultures from at least two different dissections.

### 2.6. Recombinant Mutant Rac1 Protein

Recombinant Rac1^V12^-GST or Rac1^N17^-GST protein was induced in* E. coli* DH5*α* with 0.5 mM isopropyl *β*-D-thiogalacto-pyranoside (4 h) [30]. After cell lysis (50 mM Tris-Cl pH 7.5, 50 mM NaCl, 5 mM MgCl_2_, and 1 mM dithiothreitol), a soluble protein fraction was obtained (20,000 ×g_max⁡_, 20 min, 4°C) and subjected to affinity chromatography on glutathione-conjugated agarose. Bound proteins were eluted (5 mM glutathione in lysis buffer), concentrated to 5–7 mg/mL in 50 mM Tris-Cl pH 7.5, 100 mM NaCl, and 5 mM MgCl_2_ (Centrifugal Devices, Millipore, Bedford, MA), and stored in 100 *μ*L aliquots at −80°C after snap-freezing. Protein preparations showed single bands on Coomassie blue-stained 10% SDS polyacrylamide gels.

### 2.7. Adenoviral Expression of Rac1 Mutants in SC Neurons

Recombinant, replication deficient adenovirus carrying genes for constitutively active Rac1 (Rac1^V12^ with N-terminal FLAG tag), dominant negative Rac1 (Rac1^N17^ with N-terminal FLAG tag), or lacZ were expressed in E7 chick SC neurons as described previously [31]. At the time of plating, dissociated SC neurons were infected with recombinant adenovirus at 200 moi (multiplicity of infection) in 300 *μ*L SC medium. Cultures were replenished with 200 *μ*L fresh SC medium after 12 h and grown for additional 48 h. At three days after infection, yields of neuronal infections generally exceeded 70% [31, 32]. Amplification of viral stocks was performed in 293 HEK cells and titers greater than 5 × 10^8^ plaque forming units per mL were routinely obtained. Viral stocks were stored at –80°C.

### 2.8. Quantitative ROS Imaging in SC Neuron Cell Bodies

Dissociated SC neurons were loaded with 10 *μ*M 2′,7′-dihydrodichlorofluorescein diacetate (DCF) or 5 *μ*M dihyroethidium (DMEM/10% FBS) for 30 min (37°C, 5% CO_2_ atmosphere), washed, and allowed to recover (15 min, DMEM/10% FBS). Cultures were switched to observation medium, overlaid with light mineral oil, transferred to the heated microscope stage (Zeiss Axiovert125S), and allowed to adapt for 15 min. Images of dissociated SC neurons (random fields of view) were acquired under phase contrast (20x Plan-Apo objective) and FITC illumination (Ex 465–495 nm, DM 505, Em 515–555) using a Peltier cooled CCD camera (Sensys, Photometrics, Tuscon, AZ) after the recovery phase (defined as basal condition), after pharmacological treatments (30 min), and after cytokine exposure (100 ng/mL TNF*α* or 100 ng/mL IL-1*β*). Hydrogen peroxide was added to all cultures following treatments to ensure proper loading. As our criteria for SC neurons, we analyzed only cells displaying a large round cell body and one neuronal process at least longer than three cell diameters. Maximum DCF fluorescence intensity per neuronal cell body was determined on a pixel-by-pixel basis following background subtraction (average background of all images at *t* = 0 min for each condition) followed by erosion (2 pixels) and overlay with the original image (Zeiss imaging analysis software KS 300). All values were normalized to the average DCF fluorescence intensity in control (initial conditions). No morphological changes of neuronal cell bodies were detectable in our assay conditions indicated by constant cell body areas. ROS measurements were performed in duplicate cultures obtained from 3 to 5 independent dissections. At least 50 SC neurons were measured in duplicate cultures of three independent dissections.

### 2.9. Quantitative ROS Imaging in Advancing SC Neuron Growth Cones

SC explants were incubated (30 min, DMEM/10% FBS) with 20 *μ*M DCF (5′-(and 6′)chloromethyl-dichlorodihydrofluorescein diacetate) and 4 *μ*M Calcein Blue (CB), an oxidation-inert fluorescence indicator. After recovery (15 min, DMEM/10% FBS), cultures were switched to observation medium and overlaid with light mineral oil. Images of advancing growth cones were acquired under phase contrast (40x Plan-Apo), FITC illumination (DCF fluorescence), and DAPI illumination (CB fluorescence) (Coolsnap*fx*) before (pre-stimulus images at *t* = 0, 2, 4 min) and after (after stimulus images, 2 min time intervals, 16 min time period) bath application of 100 ng/mL TNF*α*, 100 ng/mL IL-1*β*, or 10 *μ*g/mL ovalbumin. As our positive loading control, following the observation period, all growth cones were exposed to 100 *μ*M hydrogen peroxide. For image analysis, DCF and CB fluorescence intensities (*F*_DCF_ and *F*_CB_) were integrated (pixel-by-pixel basis) over the growth cone area for each growth cone observed at each time point (^Int^ *F*_DCF,*t*_ and ^Int^ *F*_CalcB,*t*_ with *t* = time interval) after background subtraction (*t* = 0 image) and the ratio of integrated DCF and CB fluorescence intensities for each growth cone at each time point was calculated (*R*_*t*_ = ^Int^ *F*_DCF,*t*_/^Int^ *F* with *t* = time interval). Next, the average ratio of integrated DCF and CB fluorescence intensities of all growth cones observed at *t* = 0 min (pre-stimulus) was calculated (^av^ *R*_0_) followed by normalization of all ratios for at *t* = 0 min (*R*_*t*_^*n*^/^av^ *R*_0_, *n* = growth cone 1, 2, to *n* for each condition). At least 15 growth cones were analyzed per condition from three different dissections to provide statistical significance (∗*P* < 0.05).

### 2.10. ROS Quantification in Growth Cone Particle Preparations

Freshly prepared GCPs (100 *μ*g in Kreb's buffer) were loaded with 20 *μ*M DCF (30 min, 4°C) in the presence of pharmacological reagents (10 *μ*M DPI, 500 *μ*M NAC), washed (14,000 ×g_max⁡_), allowed to recover (10 min, 4°C) with pharmacological reagents present, and then exposed to 100 or 200 ng/mL TNF*α* (45 min, 4°C). For lysis, GCPs were resuspended in 2% SDS, 10 mM Tris-Cl pH 7.5, 10 mM NaF, 5 mM dithiothreitol, and 2 mM EGTA; sonicated; and cleared by centrifugation (14,000 ×g_max⁡_, 5 min). Total DCF fluorescence intensity was measured (100 *μ*L aliquots, black 96 well plates) using a Beckman Coulter Multimode DTX 880 microplate reader (495 nm excitation filter, 525 emission filter). All data were adjusted to total soluble protein concentration (BCA assay) and normalized to control condition to account for unspecific fluorescence and/or autofluorescence artifact. For all conditions, measurements were obtained in duplicates from three different GCP preparations.

### 2.11. Indirect Immunocytochemistry

Dissociated SC neurons were fixed (4% paraformaldehyde, 10 mM MES pH 6.1, 138 mM KCl, 3 mM MgCl_2_, 2 mM EGTA, 15 min, RT) followed by three washes with TBS (20 mM Tris-Cl pH 7.5, 150 mM NaCl). After blocking (30 min), cultures were incubated (2 h, RT) with primary antibodies (4 *μ*g/mL, 2 mg/mL BSA in TBS) against cytokine receptors or MAP kinases, rinsed with TBS (3 times, 15 min each), and incubated with respective secondary antibodies. Cultures were transferred to 60% glycerol/PBS. Images were acquired under FITC illumination on a Radiance 2000 confocal microscope (Bio Rad).

### 2.12. Actin Filament Quantification

To visualize actin filaments, SC neurons were fixed and permeabilized (0.5% Triton X-100, 15 min). After rinsing (0.1% Triton X-100 in TBS), cultures were incubated (20 min) with rhodamine-conjugated phalloidin (1 : 10 in 1% Triton X-100 in TBS, Cytoskeleton Inc., Denver, CO), washed, and stored in 60% glycerol (4°C) until inspection. Images were acquired (40x oil, Plan Fluor) using a Zeiss LSM 510 confocal microscope equipped with a HeNe laser and an Argon laser. For each condition, 60 randomly selected SC neuron growth cones were scored for the presence of at least one large lamellipodia-like structure (three dissections, *n* = 180) and the percentage of responding growth cones determined.

### 2.13. Plasma Membrane Translocation of p67^phox^

Freshly prepared GCPs (100 *μ*g per sample) were treated with methyl-*β*-cyclodextrin (0.1%) or buffer (30 min, 4°C) and then exposed to 200 ng/mL TNF*α* (1 h, 4°C), whereas cultures of E10 forebrain neurons were subjected to 200 nM PMA (1 h, 37°C). GCPs (centrifugation) or forebrain neurons (scraping) were transferred into a 0.33 M sucrose buffer (20 mM Tris-HCL pH 8.0, 2 mM EDTA, 0.5 EGTA, 2 mM AEBSF, and 25 *μ*g/mL Leupeptin), lysed by sonication, and centrifuged (25,000 ×g_max⁡_, 15 min) to obtain an enriched plasma membrane fraction (pellet). Pellets were resuspended in sucrose buffer containing 1% Triton X-100 and 0.01% saponin (4°C, 15 min) or in 20 mM Tris-HCL pH 8.0, 2 mM EDTA, 0.5 mM EGTA, and 2 mM AEBSF with regard to gel electrophoresis or ELISA analysis, respectively. Suspensions were centrifuged (25,000 ×g_max⁡_, 15 min, 4°C), membrane protein was recovered as the supernatant, and total protein concentration was determined. For ELISA, aliquots of plasma membrane protein (20 *μ*g/mL, TBS/1% Triton-X-100) were incubated (12 h, 25°C) in 96-well high protein absorbent plates (Falcon). After blocking with BSA (5% w/v, TBS, 1% Trition-X-100, 1 h), wells were incubated (overnight, 4°C) with a polyclonal rabbit anti-p67^phox^ (3 *μ*g/mL), rinsed (TBS/1% Triton-X-100), and incubated with a goat anti-rabbit IgG antibody conjugated to HRP (1 : 2000, 45 min, 25°C). After three consecutive washes, wells were supplemented with 100 *μ*L TMB (tetramethylbenzidine) and absorbance at 620 nm was measured 10 min later (Beckman Coulter Multimode DTX 880 microplate reader. All values were adjusted to total membrane protein and normalized to control conditions (duplicates, three independent experiments).

### 2.14. Gel Electrophoresis and Western Blotting

SC tissue, SC neuron cultures, or GDPs were solubilized in 2% SDS, 10 mM Tris-Cl pH 7.5, 10 mM NaF, 5 mM dithiothreitol, and 2 mM EGTA and sonicated, and a total soluble protein fraction was obtained by centrifugation (supernatant, 14,000 ×g_max⁡_, 5 min). Fractions of total plasma membrane proteins were obtained as described above. Samples containing 20 *μ*g of total soluble protein in Laemmli buffer were subjected to 10% SDS gel electrophoresis followed by western blotting onto PVDF membranes (Bio-Rad, Hercules, CA) [33, 34]. Membranes were blocked (2% nonfat dry milk, TBS pH 8.5, overnight, 4°C), washed (TBS/0.05% Tween 20/2% BSA), and incubated with polyclonal antibodies against cytokine receptor, MAP kinase, phospho MAP kinase, NOX2, p47^phox^, or p67^phox^ (1 h, 4 *μ*g/mL, in TBS/0.05% Tween 20/2% BSA). Membranes were washed (TBS/0.05% Tween 20/2% BSA) and incubated (1 h, RT) with the respective secondary, alkaline phosphatase-conjugated IgG (1 : 10,000, Sigma, St. Louis, MO) followed by colorimetric development (NBT/BCIP one step, Thermo Scientific, Rockford, IL). The specificity of antibodies was verified in lysates of HeLa cells, RAW264 cells, and SH-SY5Y cells (including blocking peptides).

### 2.15. Statistical Analysis

One-way ANOVA analysis and a Kruskal-Wallis test were employed for comparisons among multiple conditions. Dunnett's *t*-test was used when comparing the means of multiple conditions with a single control. All statistical values are given as SEM with a significance of *P < 0.05 unless indicated otherwise. Measurements were obtained in duplicates from 3 to 5 separate experiments unless stated otherwise.

## 3. Results

### 3.1. TNF*α* and IL-1*β* Paralyze Growth Cone Motility and Induce Growth Cone Collapse

**Figure 1:**
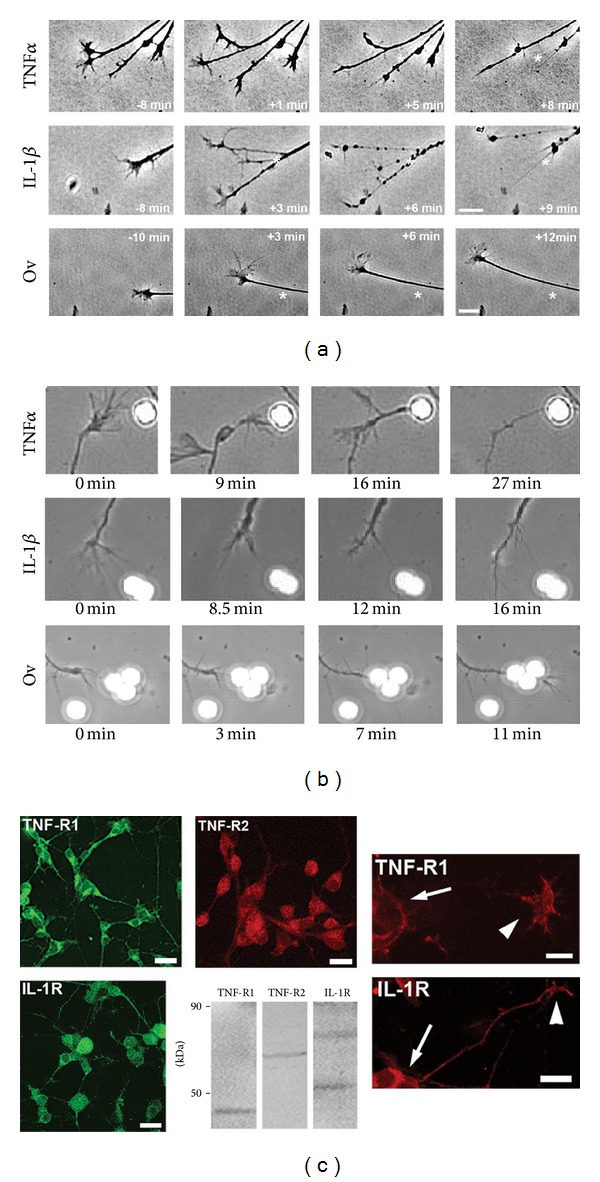
TNF*α* and IL-1*β* impair motility and morphology of neuronal growth cones. (a) Acute exposure of dissociated E7 SC neurons (laminin) to 100 ng/mL TNF*α* (top panel) or IL-1*β* (middle panel) provoked paralysis of growth cone advance and rapid degeneration of the morphology of growth cones and neurites as opposed to 10 *μ*g/mL ovalbumin (bottom panel). Stars indicate location of the growth cone/neurite border with respect to first image in each panel, respectively (scale bar = 10 *μ*m). (b) To restrict cytokine exposure exclusively to advancing growth cones, polystyrene beads (2.5 × 10^5^ beads/mL, 4 *μ*m in diameter) coated with TNF*α* or IL-1*β* were applied to SC neuron cultures and growth cone-bead encounters observed under phase contrast (63x oil, phase contrast). Following physical contact of growth cones to cytokine-coated beads (TNF*α*—top panel, IL-1*β*—middle panel), growth cone motility ceased followed by the progressive degeneration of growth cone morphology upon reaching complete collapse. Contact with ovalbumin-coated beads had no influence on growth cone morphology and advance (bottom panel). (c) E7 SC neurons grown on laminin for 2 days were fixed in paraformaldehyde. Cytokine receptors were revealed by indirect immunocytochemistry and analyzed by confocal microscopy (Zeiss LSM510, 40x oil, NA 1.30). TNF*α* receptor 1 (TNF-R1) and IL-1*β* receptor (IL-1R) were expressed on cell bodies (arrows) and neurites were expressed as well as on growth cones and filopodia (arrowheads). In contrast, TNF*α* receptor 2 (TNF-R2) expression was restricted to cell bodies. (Scale bars: upper panel, 20 *μ*m; lower panel 10 *μ*m). Western blots of whole spinal cord (E7 chick) extracts revealed immunoreactivity (stars) against avian cytokine receptors TNF-R1 (48 kDa), TNF-R2 (70 kDa), and IL-1R (76 kDa) as determined in spinal cord lysates (50 *μ*g total protein per lane).

Recent reports detailed that TNF*α* exerts morphogenetic functions (rearrangements of the actin cytoskeleton) without induction of apoptosis [7]. We examined whether TNF*α* or IL-1*β* has the potency to alter motility of neuronal growth cones, an actin-cytoskeleton driven mechanism. Live-video, phase contrast microscopy of advancing SC neuron growth cones revealed that an acute exposure to TNF*α* or IL-1*β* paralyzed growth cone motility and also caused the collapse of growth cone morphology accompanied by neurite beading and retraction compared to control (ovalbumin) (Figure 1(a)). To demonstrate a direct effect of cytokines on growth cones, we utilized polystyrene beads (4 *μ*m in diameter) covalently coated with TNF*α*, IL-1*β*, or ovalbumin (control) to restrict contact between neurons and cytokines exclusively to advancing growth cones [35]. Growth cones encountering cytokine-coated beads displayed a series of stereotypic changes in behavior ultimately resulting in growth cone collapse (Figure 1(b)). After establishing long-lasting adhesion contacts with filopodia, growth cones entered a phase characterized by highly mobile, lamellipodia-like structures, which were nonproductive for advance, followed by a collapse of morphology (TNF*α*: 78 ± 4%, *n* = 22 and IL-1*β*: 83 ± 7%, *n* = 18 of observed growth cones, resp.). None of these growth cone responses occurred upon contact with ovalbumin-coated beads. Cytokine receptors were expressed on SC neurons and their processes (Figure 1(c)). Whole SC tissue extracts (western blotting) exhibited immunoreactivity against TNF receptor 1 (TNF-R1, apparent MW = 48 kDa), TNF receptor 2 (TNF-R2, apparent MW = 70 kDa), and IL-1*β* receptor type 1 (IL-1R, apparent MW = 76 kDa) in accordance with previous reports [36]. Expression of TNF-R1 and IL-1R was found on neuronal cell bodies, neurites, as well as growth cones; however, TNF-R2 expression was predominantly localized to neuronal cell bodies with virtually no expression on neurites or growth cones.

**Figure 2:**
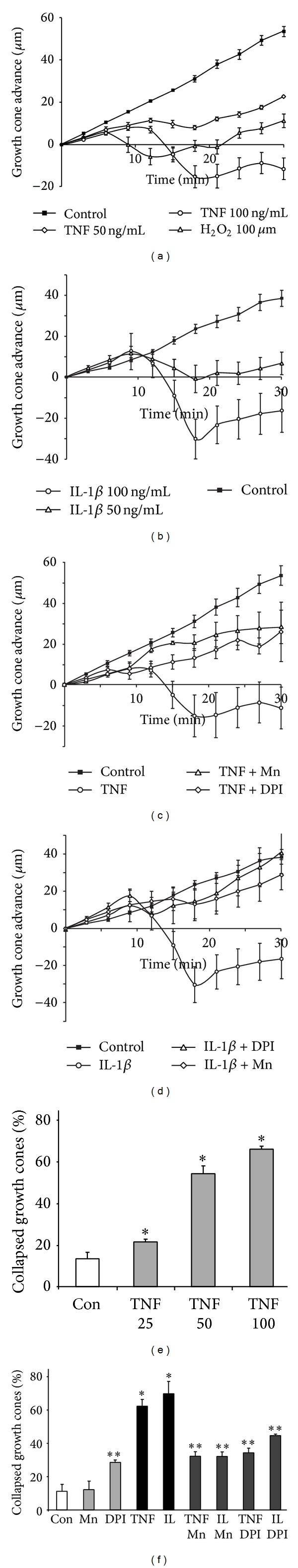
TNF*α* and IL-1*β* provoke a redox-sensitive collapse of growth cone motility and morphology. Advancing growth cones in SC neurons cultures (laminin) were randomly selected and images were acquired at 3 min time intervals (20x magnification, phase contrast) in the presence of 10 *μ*M MnTBAP, 2 *μ*M DPI, or PBS (equal volume) before and after acute exposure (*t* = 6 min) to TNF*α* (50 or 100 ng/mL), IL-1*β* (50 or 100 ng/mL), or ovalbumin (10 *μ*g/mL). Growth cone advance was measured as the extension of the growth cone/neurite boundary (*μ*m) per time interval and plotted against time (min) with slopes indicating growth rates. (a and b) Growth cones ceased motility and advance within minutes upon exposure to cytokines (open diamonds, open circles) compared to a steady growth cone advance under control (ovalbumin, closed squares). Growth cones exposed to 100 *μ*M H_2_O_2_ (open triangles) mostly responded with paralysis, yet slow recovery was measured. In the presence of 50 ng/mL cytokines (open diamonds), growth cones resumed advance after a lag phase however at much slower growth rates, whereas no recovery was detected at concentrations of 100 ng/mL TNF*α* or IL-1*β* (open circles). (c and d) ROS scavenging with 5 *μ*M MnTBAP (open triangles) or NOX inhibition with 2 *μ*M DPI (open diamonds) rescued growth cone advance upon acute exposure to 100 ng/mL TNF*α* (c) or 100 ng/mL IL-1*β* (d), respectively (open circles). All data were obtained from at least three different dissections (duplicate cultures each, >30 growth cones total) with error bars representing SEM. (e) TNF*α* elicited a dose-dependent growth cone collapse at concentrations higher than 50 ng/mL. Growth cones with collapsed morphology were quantified (random fields of view) 30 min after application to allow for possible recovery of morphology. (f) Preincubation of SC neuron culture either with 10 *μ*M MnTBAP or with 2 *μ*M DPI provided significant protection against growth cone collapse in the presence of 100 ng/mL TNF*α* or 100 ng/mL IL-1*β* (dark grey bars; **P < 0.05) as opposed to cytokines alone (black bars), which caused substantial growth cone collapse (dark bar, *P < 0.05) compared to control (open bar). A presence of 10 *μ*M MnTBAP (light grey bar, Mn) had no effect on basal levels of collapsed growth cones, whereas 2 *μ*M DPI increased the percentage of collapse growth cones. All data (e and f) were obtained from at least three different dissections (duplicate cultures each). Error bars represent ±SEM.

Next, we quantified growth cones advance (the spatial displacement of the growth cone/neurite boundary over time) in SC explant cultures upon acute exposure to TNF*α*, IL-1*β*, or ovalbumin (Ov, control) (Figure 2). Both TNF*α* and IL-1*β* caused a rapid, dose-dependent paralysis of growth cone advance and even neurite retraction within less than 10 minutes of addition compared to Ov (Figures 2(a) and 2(b)). Acute bath application of 100 ng/mL TNF*α* or IL-1*β* reduced the percentage of advancing growth cones by 83 ± 6% (*n* = 48, *P < 0.05) compared to bath application of 10 *μ*g/mL Ov. In contrast, a considerable fraction of growth cones resumed advance following bath application of 50 ng/mL TNF*α* or IL-1*β* (TNF*α*: 42 ± 14%, *n* = 24, *P < 0.05 and IL-1*β*: 30 ± 8% *n* = 20, *P < 0.05) however at much slower growth rates (31 ± 6 *μ*m/h and 21 ± 5 *μ*m/h, resp.) compared to growth rates prior to cytokines application (TNF*α*: 79 ± 8 *μ*m/h, *n* = 22 and IL-1*β*: 89 ± 9 *μ*m/h, *n* = 26). As expected, application of 10 *μ*g/mL Ov had no effect on growth cone advance (before application: 108 ± 10 *μ*m/h and after application: 113 ± 6 *μ*m/h). According to reports and our own findings, we tested whether ROS intermediates were implicated in cytokine-mediated growth cone paralysis and collapse. Scavenging ROS with 10 *μ*M MnTBAP or inhibition of NADPH oxidase (NOX) activities with 2 *μ*M DPI both protected growth cone advance upon exposure to 100 ng/mL TNF*α* (69 ± 1%, *n* = 26 and 71 ± 10%, *n* = 38 of advancing growth cones, resp.) albeit with decreased growth rates (96 ± 9 *μ*m/h, 17% reduction and 82 ± 9 *μ*m/h, 26% reduction, resp.) compared to 10 *μ*g/mL Ov (Figure 2(c)). Growth cone advance was not affected by an addition of MnTBAP (124 ± 10 *μ*m/h) or DPI (98 ± 13 *μ*m/h) in the presence of ovalbumin during the period of observation. Growth cone advance in the presence of 100 ng/mL IL-1*β* was also rescued by 10 *μ*M MnTBAP or 2 *μ*M DPI (77 ± 12%, *n* = 38 and 69 ± 6%, *n* = 50, resp.) (Figure 2(d)). Exogenous addition of ROS to growth cones (100 *μ*M hydrogen peroxide) also reduced the percentage of advancing growth cones (44 ± 2%, *n* = 42, *P < 0.05).

Both cytokines also elicited a dose-dependent growth cone collapse quantified 30 min after addition as shown for TNF*α* (25 ng/mL: 16 ± 3%, *n* = 135; 50 ng/mL: 59 ± 4%, ∗*P* < 0.05, *n* = 125; 100 ng/mL: 71 ± 8%, *P < 0.05, *n* = 122) compared to 10 *μ*g/mL Ov (13 ± 3%, *n* = 69) (Figure 2(e)). Moreover, scavenging ROS (10 *μ*M MnTBAP) or inhibiting NOX (2 *μ*M DPI) (32 ± 4%, *n* = 100 and 34 ± 4%, *n* = 99, resp., ∗∗*P* < 0.05) protected growth cone morphology from the presence of 100 ng/mL TNF*α* as opposed to the absence of pharmacological agents (63 ± 7%,  *n* = 87, ∗*P* < 0.05) (Figure 2(f)). Similarly, MnTBAP (31 ± 3%, *n* = 156) and DPI (44 ± 1%, *n* = 186) also negated IL-1*β*-induced growth cone collapse (69 ± 8%, *n* = 196, *P < 0.05). The percentage of collapsed growth cone remained unaltered by the presence of 10 *μ*M MnTBAP (11 ± 6%, *n* = 99), yet it did significantly increase with 2 *μ*M DPI (27 ± 1%, *n* = 63). Taken together, these data revealed that TNF*α* and IL-1*β* caused rapid degeneration of growth cone morphology and complete loss of motility through a redox-dependent mechanism. The rapid response of growth cones to cytokines together with the expression of cytokines receptors on growth cones strongly suggested a direct mechanism as opposed to indirect effects through neurodegenerative processes originating in neuronal cell bodies.

### 3.2. A Cytokine-Activated NADPH Oxidase Generates ROS in Growth Cones and Cell Bodies of SC Neurons

**Figure 3:**
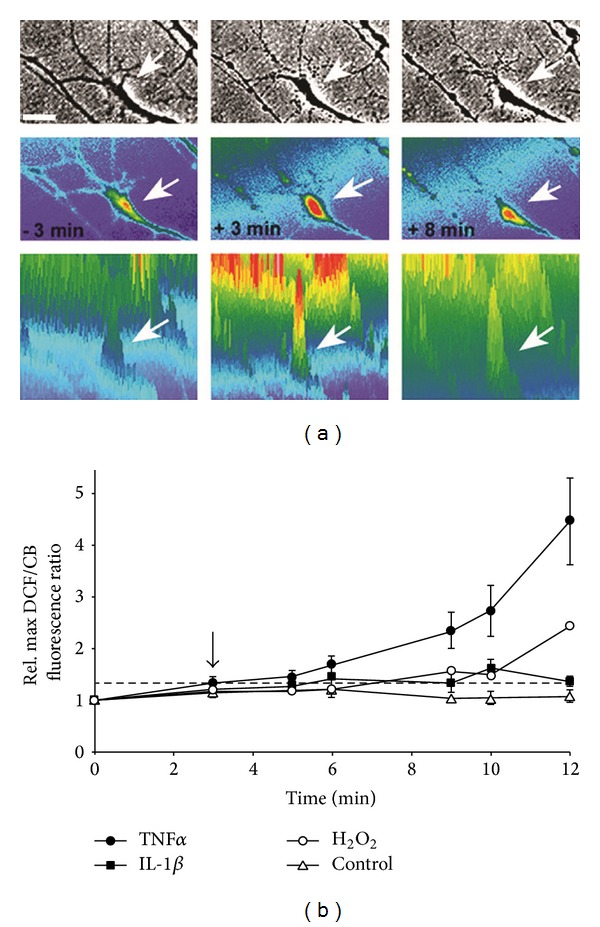
TNF*α* stimulates ROS formation in advancing growth cones. ROS formation in advancing SC neuron growth cones was revealed by ratiometric fluorescence imaging. Dissociated E7 chick SC neurons (laminin) were loaded (30 min) with the oxidation-sensitive fluorescent indicator 2′,7′-dihydrodichlorofluorescein (10 *μ*M DCF) and the oxidation-inert fluorescent indicator Calcein blue (4 *μ*M CB). Images of randomly selected growth cones were acquired under FITC fluorescence illumination (DCF), DAPI fluorescence illumination (CB), and phase contrast at short time intervals before and after addition of stimuli (40x, oil, identical parameters). (a) Intracellular ROS formation is represented by a heat spectrum ranging from blue (basal ROS levels) to red (increased ROS levels). Growth cone morphology (arrow) disintegrated over a time period of 11 minutes upon acute exposure to 100 ng/mL TNF*α* (phase images, upper panel). Ratio imaging (middle panel) revealed a sharp increase in ROS production (*t* = +3 min) shortly after TNF*α* exposure that persisted (*t* = +8 min) compared to basal levels (*t* = −3 min) further illustrated by profile images of ratiometric images (lower panel) (Scale bar = 5 *μ*m). Note the loss of filopodia and lamellipodia and the contraction of the growth cone body (last panel) were preceded by an increase in ROS. (b) ROS formation in advancing SC neuron growth cones was quantified by ratiometric fluorescence imaging. Maximum DCF and CB fluorescence intensities per growth cone area were determined on a pixel-by-pixel basis and DCF/CB ratios calculated for each condition and time point. All DCF/CB ratios were normalized (average DCF/CB ratio at *t* = 0 min) and plotted against time. Exposure to 100 ng/mL TNF*α* (filled circles, *n* = 14), 100 ng/mL IL-1*β* (filled squares, *n* = 10), or 100 *μ*M hydrogen peroxide (positive control, open circles, *n* = 11) at *t* = 3 min (arrow) elicited a significant ROS formation (stippled line = significance threshold) in advancing growth cones within less than 5 min upon addition compared to 10 *μ*g/mL ovalbumin (negative control, open triangles, *n* = 22).

To determine the generation of intracellular ROS in advancing growth cones exposed to cytokines, we employed quantitative ratiometric fluorescence analysis utilizing the oxidation-sensitive fluorescent indicator 2′,7′-dihydrodichlorofluorescein (DCF, 10 *μ*M) and the redox inert fluorescence indicator Calcein Blue (CB, 4 *μ*M). Ratiometric analysis distinguishes changes in DCF fluorescence due to ROS formation from dilution/concentration effects simply due to rapid changes in growth cone morphology. Bath application of 100 ng/mL TNF*α* or 100 ng/mL IL-1*β* elicited a rise in the relative maximum DCF/CB fluorescence ratio indicative of the formation of ROS (Figure 3). As shown in Figure 3(a) (false colored DCF/CB ratio images), ROS formation was sustained and preceded loss of morphology of the advancing growth cone reflected by atrophy and beading of filopodia, condensation of growth cone body, and retraction. Quantitative analysis demonstrated a significant and sustained ROS formation in advancing growth cones upon exposure to TNF*α* (100 ng/mL, *n* = 14), IL-1*β* (100 ng/mL, *n* = 10), or 100 *μ*M hydrogen peroxide (positive control, *n* = 11) compared to 10 *μ*g/mL ovalbumin (negative control, *n* = 22) (Figure 3(b)). Neither loading with DCF (131 ± 18 *μ*m/h, *n* > 15) nor loading with CB (118 ± 14 *μ*m/h, *n* > 15) at the concentrations used affected growth cone advance per se compared to unloaded control (113 ± 9 *μ*m/h).

**Figure 4:**
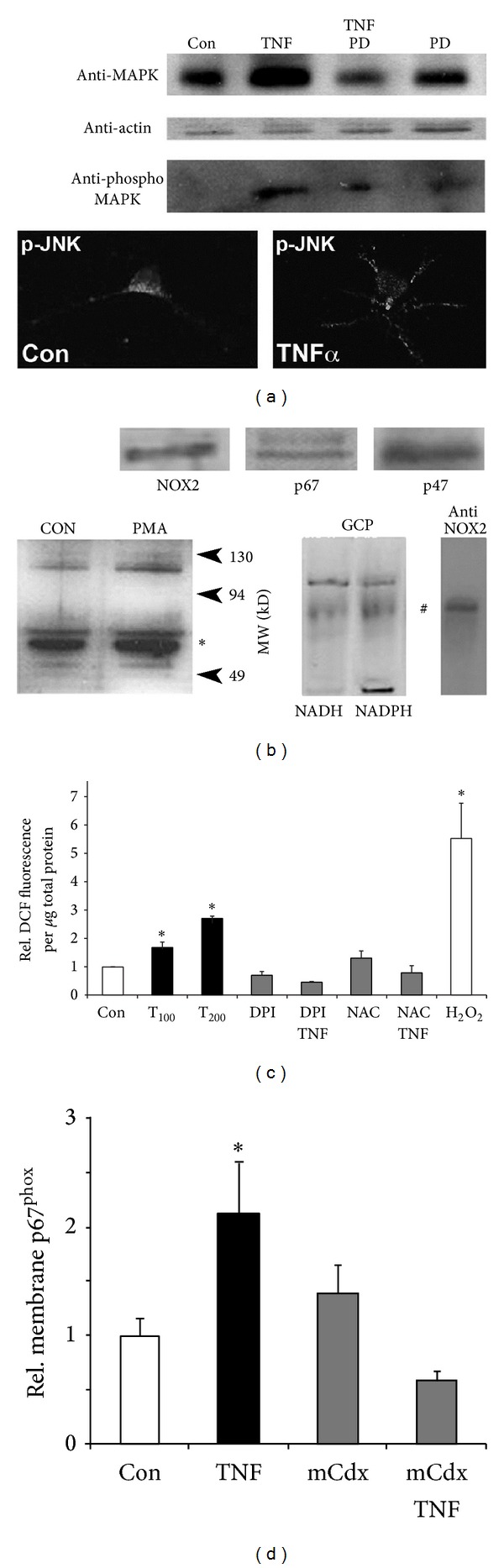
NADPH oxidase mediates ROS formation in growth cones exposed to TNF*α*. (a) Exposure of freshly isolated growth cone particles (GCPs) to TNF*α* activates MAP kinase. GCPs were plated on laminin in Krebs buffer (2 h), washed, and incubated with the MAP kinase inhibitors PD98059 (25 *μ*M, 20 min) or PBS (equivalent volume) prior to an addition of 100 ng/mL TNF*α*. Attached GCPs were lysed and equal amounts of total protein were subjected to SDS gel electrophoresis (10%) followed by western blotting and detection (chemiluminescence) of MAP kinase (top panel) and phospho-MAP kinase immunoreactivity (bottom panel). Actin served as a loading control (middle panel). Exposure of GCPs to TNF*α* stimulated MAP kinase activity (increased phospho-MAP kinase), which was negated by PD98059. Also, TNF*α* treatment enhanced expression of MAP kinase (increases in total MAP kinase), a response apparently diminished by a presence of PD98059. Confocal images acquired (63x, oil) of cultured chick forebrain neurons (laminin, 24 h) exposed to 100 ng/mL of TNF*α* (TNF*α*) revealed phospho-JNK immunoreactivity (white signal) as a discrete, punctate pattern in cell bodies and neuronal process in contrast to the homogenous appearance in neuronal soma under control conditions (PBS, equivalent volume). Cultures were fixed with 4% paraformaldehyde and immunostained against phospho-JNK. (b) Neuronal growth cones contain a functional NADPH oxidase activity. Lysates obtained from SC neuron cultures revealed immunoreactivity against the large membrane subunit NOX2 and the cytosolic subunits p67^phox^ and p47^phox^. Incubation of chick (E7) forebrain neurons with the NOX activator PMA (400 ng/mL) increased plasma membrane association of the cytosolic subunit p67^phox^ (∗) compared to controls (Con) indicative for the formation of the NOX multiprotein complex. Native gel electrophoresis, combined with in-gel NBT staining, of freshly isolated growth cone particles (GCPs) obtained from E10–12 chick forebrain demonstrated one NADPH oxidoreductases activity (#) colocalizing with NOX2 immunoreactivity. (c) GCPs were loaded with 10 *μ*M DCF (30 min) in the presence of 2 *μ*M DPI (NOX inhibitor), 500 *μ*M NAC (antioxidant), or PBS (mock, equivalent volume) prior to addition of TNF*α* (45 min). DCF fluorescence was determined in GCP lysates, adjusted to total soluble protein, and normalized to control (Con, open bar). TNF*α* stimulated a significant, dose-dependent ROS formation in GCPs (T_100_ = 100 ng/mL, T_200_ = 200 ng/mL, *P < 0.05), which was abolished by DPI or NAC. Peroxide (H_2_O_2_) served as a positive control. (d) Plasma membrane association of p67^phox^ normalized to control conditions was quantified (western blotting) using plasma membrane-enriched fractions. Exposure of GCPs to 200 ng/mL TNF*α* significantly increased the relative plasma membrane association of p67^phox^ (TNF, *P < 0.05) compared to control (Con) indicating the formation of a functional NOX complex. Preincubation of GCPs with methyl-*β*-cyclodextrin (0.1%) negated plasma membrane translocation of p67^phox^ in the presence of TNF*α* (mCdx-TNF). All data represent SEM from at least two experiments (triplicate conditions each) with *P < 0.05.

**Figure 5:**
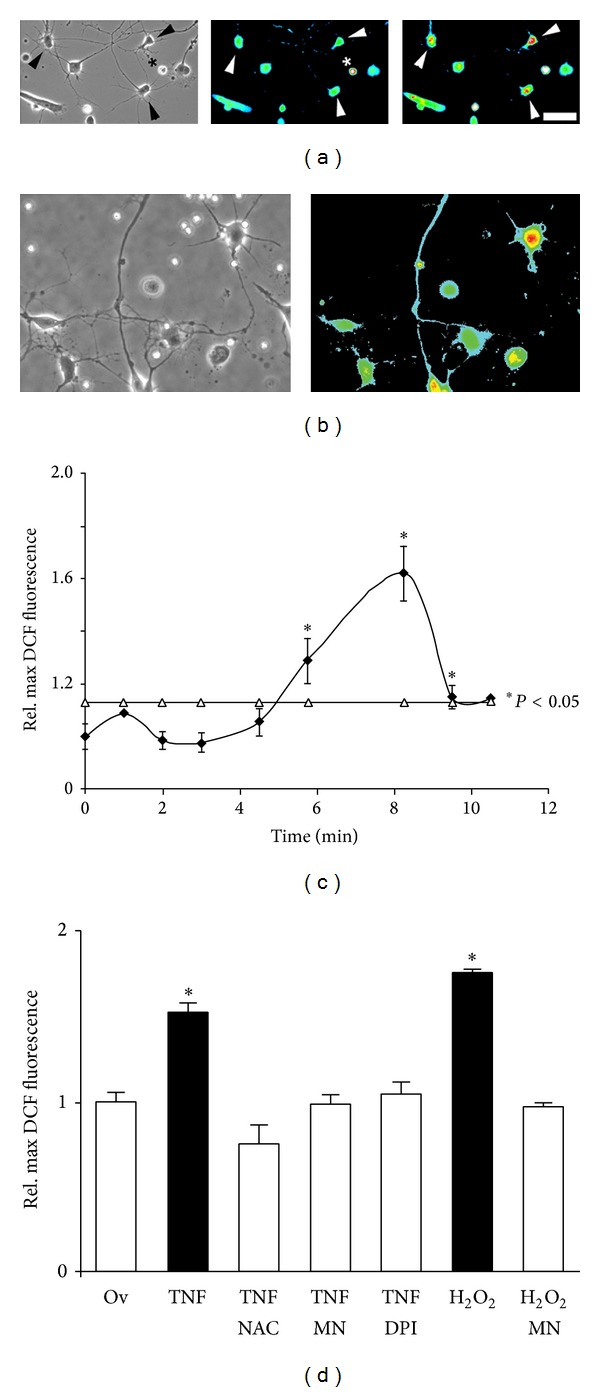
Cytokines elicit ROS formation in cell bodies of SC neurons. Dissociated E7 SC neurons were loaded with 10 *μ*M DCF (30 min), allowed to recover for 15 min, and then incubated (45 min) with NAC, MnTBAP, DPI, or PBS (equal volume) prior to cytokines exposure. Images were acquired at identical parameters (20x). (a) DCF-loaded SC neurons exhibited a distinct neuronal morphology (arrowheads) extending one long and several shorter neuronal processes (left panel, phase contrast image). Note cultures are virtually free of nonneuronal cells. Under control conditions, SC neuron cell bodies displayed basal levels of ROS formation (middle panel, arrowheads). Degenerating cells (star) exhibit very high fluorescence intensity in the absence of any stimuli. Addition of 100 ng/mL TNF*α* stimulated a robust increase in intracellular ROS (right panel, arrowheads, 8 min after addition). Fluorescence images were false colored (heat spectrum) with fluorescence intensities increasing from blue (basal ROS levels) to red (high ROS levels). (b) TNF*α*-coated polystyrene beads increased DCF fluorescence intensity in SC neurons indicative of ROS formation. Although qualitative in nature, SC neurons with multiple bead contacts (arrow head) as opposed to single bead contact (asterisk) displayed more intense DCF fluorescence. (c) Maximum DCF fluorescence intensities per neuronal cell body were determined on a pixel-by-pixel basis after background subtraction over time and all values normalized to the average maximum DCF fluorescence intensity under control conditions at* t* = 0 min (relative maximum DCF fluorescence intensity). SC neurons responded with a transient increase in ROS formation in cell bodies (16 ± 2 cells per time interval) upon exposure to 100 ng/mL TNF*α* (2.5) min (threshold of statistical significance *P < 0.01 shown as open triangles). (d) 100 ng/mL TNF*α* stimulated a significant increase in ROS formation (*P < 0.01), which was negated by the presence of ROS scavengers (TNF*α*-NAC 2 mM or TNF*α*-MnTBAP 10 *μ*M, resp.) or NOX inhibitor (TNF*α*-DPI 5 *μ*M) to levels indistinguishable from 10 *μ*g/mL ovalbumin (Ov), our control. As a control for DCF loading, 200 *μ*M hydrogen peroxide (H_2_O_2_) greatly increased relative maximum DCF fluorescence (*P < 0.01), which was suppressed in the presence of 10 *μ*M MnTBAP (H_2_O_2_-Mn). All measurements were obtained from at least three independent dissections (duplicate cultures each).

**Figure 6:**
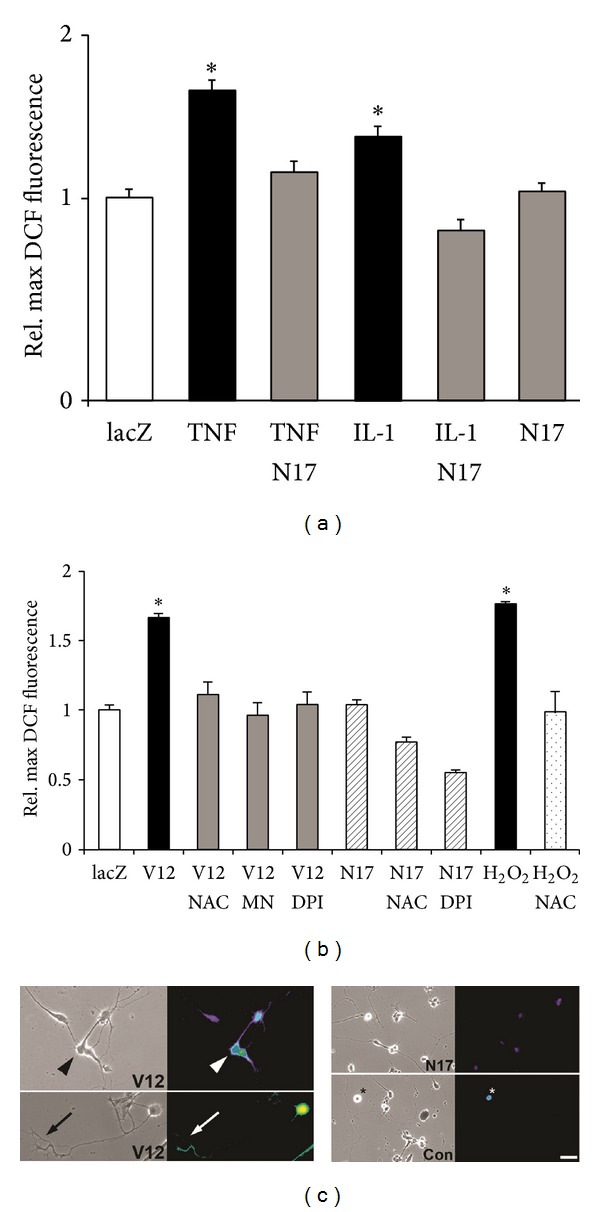
TNF*α* and IL-1*β* stimulate a Rac1-mediated ROS formation in SC neurons. Dissociated SC neurons grown on laminin were infected with recombinant, replication deficient adenovirus (200 moi, infection at the time of plating) carrying FLAG-tagged constitutively active Rac1^V12^, FLAG-tagged dominant negative Rac1^N17^, or lacZ. Three days after infection, SC neurons were loaded with 10 *μ*M DCF (30 min), incubated with NAC, MnTBAP, or DPI, and exposed to cytokines (100 ng/mL). Random images were acquired (20x) under FITC fluorescence illumination, relative maximum DCF fluorescence intensity was quantified, and all values normalized to lacZ-expressing SC neurons exposed to 10 *μ*g/mL Ovalbumin (lacZ), our control. (a) Expression of Rac^N17^ completely abolished ROS formation in SC neurons upon exposure to 100 ng/mL TNF*α* or IL-1*β* (TNF*α*-N17 and IL-1*β*-N17, resp.) compared to lacZ-expressing SC neurons (TNF*α* and IL-1*β*, resp., *P < 0.01) without altering basal levels of ROS formation (N17). (b) Expression of Rac1^V12^ was sufficient to induce ROS formation in SC neurons, which was negated by 2 mM NAC, 10 *μ*M MnTBAP, or 10 *μ*M DPI. Peroxide (200 *μ*Mol/L H_2_O_2_) served as a DCF loading control. (c) DCF-loaded SC neurons expressing Rac^V12^ (V12) revealed increases in ROS formation both in cell bodies (arrowhead) as well as in neuronal growth cones (arrow) and distal neurites (left panel). In contrast, SC neurons expressing Rac^N17^ (N17) exhibited basal DCF fluorescence intensity compared to lacZ (Con) expressing SC neurons (right panel). Note the intense DCF fluorescence in a degenerating cell (asterisk). A heat spectrum ranging from blue (basal ROS levels) to red (increased ROS levels) indicates ROS production (scale bar = 40 *μ*m).

To examine NOX-dependent ROS production in neuronal growth cones, we utilized freshly isolated growth cone particle preparations (GCPs) obtained from chick E10 forebrain neurons, which are capable of showing complex responses to extrinsic stimuli [37–39]. As shown in Figure 4(a), addition of 100 ng/mL TNF*α* to laminin-adherent GCPs resulted in MAP kinase and JNK activation, which was corroborated in cultured forebrain neurons. No basal MAP kinase activity (phosphor MAP kinase) was detectable in GCPs under control conditions possibly due a lack of growth factors (serum free plating conditions). Indicative for the formation of a functional NOX multiprotein complex, the NOX activator PMA (200 ng/mL) significantly increased the relative plasma membrane association of p67^phox^ in chick forebrain neurons (Figure 4(b)). Moreover, native gel electrophoresis and NBT staining revealed several NADPH oxidoreductase activities in GCPs, one colocalizing with NOX2 immunoreactivity. The large membrane subunit NOX2 and the cytosolic subunits p67^phox^ and p47^phox^ were also detected in lysates of SC neurons (Figure 4(b)). Addition of TNF*α* (100 ng/mL) to DCF-loaded GCPs stimulated a significant, dose-dependent increase in ROS formation, which was negated by the NOX inhibitor DPI (5 *μ*M) or the ROS scavenger NAC (1 mM) (Figure 4(c)). The small spatial dimensions of plated GCPs prevented ROS quantification utilizing microscopic imaging. Therefore, we resorted to a protocol involving lysis as previously established [14]. Moreover, plasma membrane association of p67^phox^ in GCPs significantly increased in response to 100 ng/mL TNF*α*, which was abolished by the lipid raft disrupter methyl-*β*-cyclodextrin, suggesting that the assembly of NOX occurred predominantly in lipid rafts (Figure 4(d)). The insignificant increase of p67^phox^ in plasma membrane fractions from methyl-*β*-cyclodextrin-treated cells could result from unspecific adsorption due to the dramatic change in membrane lipid composition. TNF*α*-coated polystyrene beads or bath application of 100 ng/mL TNF*α* to DCF-loaded SC neurons stimulated a transient increase in DCF fluorescence intensity in neuronal cell bodies (Figures 5(a) and 5(b)). Cytokine-stimulated ROS formation was dose-dependent with significant stimulation at concentrations higher than 50 ng/mL and saturation at 100 ng/mL (data not shown). Maximum ROS formation (1.62 ± 0.1, *P < 0.01) in SC neuron cell bodies occurred 5.3 ± 0.8 min following bath application of 100 ng/mL TNF*α* lasting for 6.4 ± 0.6 min (16 ± 2 cells per time interval) (Figure 5(c)). 100 ng/mL IL-1*β* also increased ROS formation (1.39 ± 0.02, *P < 0.01) within 5 ± 0.7 min upon application and lasted for 7.8 ± 1.4 min (*n* = 20 ± 2 cells per time interval). Scavenging ROS with 2 mM NAC or 10 *μ*M MnTBAP both suppressed TNF*α*-stimulated ROS formation (0.75 ± 0.11; *n* = 29 and 0.99 ± 0.05; *n* = 65, resp.) to levels indistinguishable from control (10 *μ*g/mL ovalbumin, 0.99 ± 0.05, *n* = 123) in contrast to TNF*α* alone (1.53 ± 0.05, *P < 0.01, *n* = 184) (Figure 5(d)). Inhibiting NOX activity (5 *μ*M DPI) also negated ROS formation (1.03 ± 0.07; *n* = 70, *P < 0.01) upon exposure to TNF*α*. Similar data were obtained upon acute addition of 100 ng/mL IL-1*β* to SC neurons (data not shown). As our positive control, 200 *μ*M hydrogen peroxide greatly increased relative maximum DCF fluorescence intensity (1.75 ± 0.02; *n* = 109, *P < 0.01), which was abolished in the presence of 10 *μ*M MnTBAP (0.97 ± 0.02; *n* = 109) (Figure 5(d)). Depleting Rac1 activity using adenoviral-mediated expression of FLAG-tagged, dominant-negative Rac1^N17^ completely suppressed TNF*α* or IL-1*β*-stimulated ROS formation (1.13 ± 0.05, *n* = 93 and 0.84 ± 0.05, *n* = 71, resp.) as opposed to lacZ expression (TNF*α*: 1.53 ± 0.05, *P < 0.05, *n* = 184, and IL-1*β*: 1.30 ± 0.05, *P < 0.05, *n* = 117, resp.) (Figure 6(a)) [31]. In contrast, expression of constitutively active Rac1^V12^ in SC neurons was sufficient to increase ROS formation (1.65 ± 0.04, *n* = 450, *P < 0.05) compared to 200 *μ*M hydrogen peroxide (1.98 ± 0.16, *n* = 60, *P < 0.05) (Figures 6(b) and 6(c)). ROS scavenging with 2 mM NAC or 10 *μ*M MnTBAP as well as NOX inhibition with 10 *μ*M DPI abolished Rac1^V12^-stimulated ROS formation (1.11 ± 0.09, *n* = 62; 0.96 ± 0.09, *n* = 109; and 1.05 ± 0.07, *n* = 138, resp.). Similar results were obtained using purified recombinant GST chimeras of Rac1^V12^ and Rac1^N17^ transfected into E7 SC neurons by trituration loading (data not shown). Expressing Rac1^N17^ alone (1.03 ± 0.04, *n* = 247) or lacZ (0.99 ± 0.03, *n* = 105), our control, had no effect on basal levels of ROS (Figure 6(c)). However addition of 2 mM NAC or 10 *μ*M DPI to Rac1^N17^-expressing SC neurons both reduced ROS formation significantly below basal levels (0.76 ± 0.04, *n* = 94 and 0.55 ± 0.02, *n* = 106, resp., *P < 0.05). These findings revealed that TNF*α* and IL-1*β* stimulate a functional assembly of a Rac1-regulated NOX2 complex in the plasma membrane of neuronal growth cones resulting in a transient increase in ROS formation.

### 3.3. TNF*α* and IL-1*β* Attenuate Neurite Outgrowth of SC Neurons in a Redox-Dependent Manner

**Figure 7:**
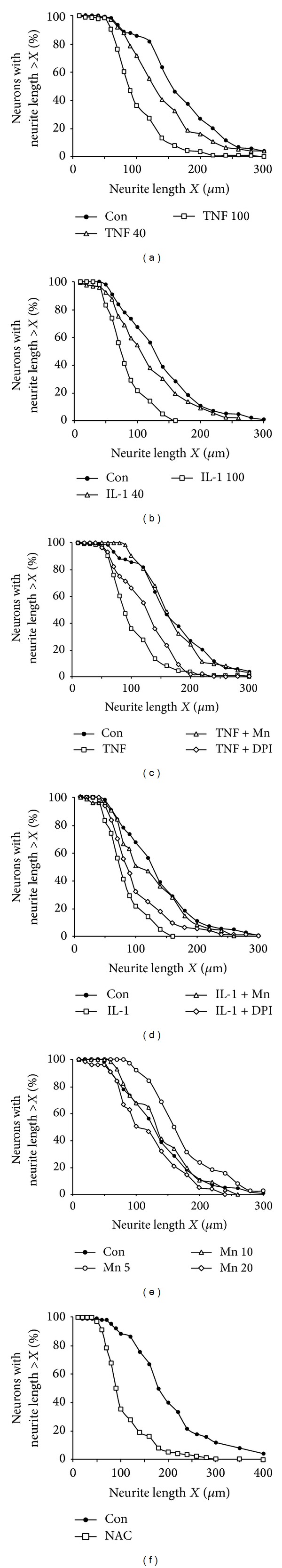
TNF*α* and IL-1*β* impede neurite outgrowth in a redox-sensitive manner. Dissociated SC neurons were grown on laminin past the onset of neurite initiation and then incubated with 10 *μ*M MnTBAP, 2 *μ*M DPI, or PBS (10 *μ*L) for 1 h prior to bath application of cytokines or ovalbumin (6 to 8 h) followed by fixation (2% glutaraldehyde). The longest neurite per neuron was measured of randomly selected neurons and the percentage of neurons with a given neurite length was plotted against neurite length. (a and b) Persistent presence of TNF*α* (a) or IL-1*β* (b) significantly reduced neurite outgrowth in a dose-dependent manner (40 ng/mL, open triangles; and 100 ng/mL, open squares) indicated by the shift of the neurite length distribution to shorter neurites compared to control (10 *μ*g/mL ovalbumin, filled circles). (c and d) Despite a continuous presence of 100 ng/mL TNF*α* ((c), open squares) or 100 ng/mL IL-1*β* ((d), open squares), scavenging ROS with 10 *μ*M MnTBAP (open triangles) rescued neurite outgrowth compared to controls (10 *μ*g/mL ovalbumin, filled circles), whereas inhibiting NOX activity with 2 *μ*M DPI (open diamonds) was only partially protective. (Neurite number measured ≥ 60 for each condition, two experiments, and duplicate cultures). (e) In the presence of 20 *μ*M MnTBAP (open diamonds), neurite outgrowth was significantly decreased compared to control (PBS, filled circles). However, concentrations of 10 *μ*M MnTBAP (open triangles) did not significantly alter neurite outgrowth and 5 *μ*M MnTBAP (open circles) on the contrary causes a significant increase in neurite length. (Neurite number measured > 75 for each condition, two experiments, and duplicate cultures). (f) Lastly, an overabundance of the radical scavenger NAC (2 mM, open squares) dramatically reduced neurite outgrowth compared to control (PBS, filled circles) indicated by the shift towards shorter neurites.

Next, we examined whether the redox-dependent impairment of growth cone advance is of consequence for neurite outgrowth. Bath application of 100 ng/mL TNF*α* or IL-1*β* attenuated neurite outgrowth in SC neuron cultures in a dose-dependent manner indicated by a shift in the distribution to shorter neurite lengths compared to 10 *μ*g/mL ovalbumin (Ov), our control (Figures 7(a) and 7(b), Table 1). Scavenging ROS (10 *μ*M MnTBAP) rescued neurite outgrowth in the presence of TNF*α* and IL-1*β*, whereas the NOX inhibitor (2 *μ*M DPI) provided only partial protection of neurite outgrowth (Figures 7(c) and 7(d)). Notably, neurite outgrowth of SC neurons on laminin exhibited inherent redox dependence even in the absence of TNF*α* or IL-1*β* (Figures 7(e) and 7(f)). In accordance with previous reports, increasing concentrations of the ROS scavenger N-acetyl-L-cysteine (2 mM NAC, 50% reduction) or 20 *μ*M MnTBAP (10% reduction) inhibited neurite outgrowth compared to control, whereas 10 *μ*M MnTBAP was ineffective [15]. However, we measured a significant increase in neurite outgrowth with 5 *μ*M MnTBAP (25% increase).

**Figure 8:**
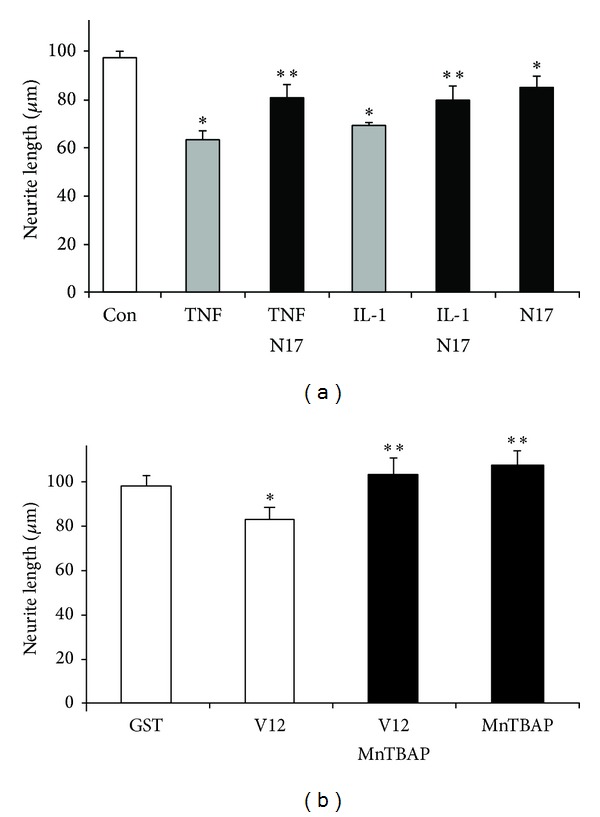
The small GTPase Rac1 mediates redox-dependent neurite outgrowth. Freshly dissected SC neurons were trituration-loaded with purified recombinant Rac1^V12^-GST, Rac1^N17^-GST, or GST only (7 mg/mL each) and grown (laminin) after the onset of neurite initiation. Cultures were supplemented with 100 ng/mL TNF*α*, 100 ng/mL IL-1*β*, or 10 *μ*g/mL ovalbumin (8 h) and the average neurite length of the longest neurite reached by 50% of SC neurons (NL_50_) for each condition quantified (*n* > 50). (a) Introduction of Rac1^N17^-GST (dominant negative mutation, black bars) significantly protected neurite outgrowth in the presence of TNF*α* (TNF*α*-N17, **P < 0.05) or IL-1*β* (IL-1*β*-N17, **P < 0.05) compared to SC neurons loaded with GST, which exhibited a great reduction in neurite length in the presence of cytokines (grey bars *P > 0.05) compared to control (ovalbumin, open bar). Not unexpected, Rac1^N17^-GST even in the absence of cytokines reduced neurite outgrowth (N17, *P < 0.05) compared to control. (b) Similarly, introduction of Rac1^V12^-GST (constitutively active mutation) significantly reduced neurite lengths (V12, *P < 0.05) compared to GST-loaded SC neurons (GST). Notably, the presence of 5 *μ*M MnTBAP increased neurite outgrowth of Rac1^V12^-GST-loaded SC neurons (V12-MnTBAP, **P < 0.05) to levels indistinguishable from neurite outgrowth of GST-loaded SC neurons treated with 5 *μ*M MnTBAP (MnTBAP, **P < 0.05) compared to control. All data were obtained from at least three different dissections (duplicate cultures each) with error bars representing.

To assess the role of Rac1 on neurite outgrowth, Rac1^V12^ (constitutively active Rac1) or Rac1^N17^ (dominant negative Rac1) was introduced as purified recombinant GST chimera into freshly dissected SC neurons by trituration loading [26] (Figure 8). Depletion of Rac1 activity (Rac1^N17^-GST) in SC neurons protected neurite outgrowth upon addition of 100 ng/mL TNF*α* (NL_50_ = 81 ± 5 *μ*m, *n* = 85, **P < 0.05) or 100 ng/mL IL-1*β* (NL_50_ = 79 ± 6 *μ*m, *n* = 68,  **P < 0.05) compared to GST-loaded SC neurons (NL_50_ = 64 ± 3 *μ*m, *n* = 75 and NL_50_ = 69 ± 2 *μ*m, *n* = 51), respectively (Figure 8(a)). However, introduction of Rac1^N17^-GST diminished neurite outgrowth even in the absence of adverse stimuli (NL_50_ = 84 ± 4 *μ*m, *n* = 132, *P < 0.05) compared to GST-loaded SC neurons (NL_50_ = 91 ± 3 *μ*m, *n* = 99) in accordance with previous findings [26, 40]. Constitutive activation of Rac1 (Rac1^V12^-GST) in SC neurons also resulted in reduction of neurite length (NL_50_ = 83 ± 5 *μ*m, *n* = 195, *P < 0.05) compared to GST-loaded SC neurons (NL_50_ = 98 ± 5 *μ*m, *n* = 112) (Figure 8(b)). Interestingly, addition of 5 *μ*M MnTBAP restored neurite outgrowth of Rac^V12^-GST-loaded SC neurons (NL_50_ = 108 ± 6, *n* = 136, **P < 0.05) to levels indistinguishable from GST-loaded SC neurons (NL_50_ = 103 ± 7 *μ*m, *n* = 103, **P < 0.05). This finding implied a role for Rac1 as a regulator of TNF*α* or IL-1*β*-stimulated oxidative stress. These findings provided evidence that attenuation of neurite outgrowth in the presence of TNF*α* or IL-1*β* required a persistent, Rac1-regulated formation of ROS. Moreover, neurite outgrowth on laminin exhibited an innate redox-sensitivity.

### 3.4. Cytokines Elicit a Redox-Dependent Reorganization of Actin Filaments in Neuronal Growth Cones

**Figure 9:**
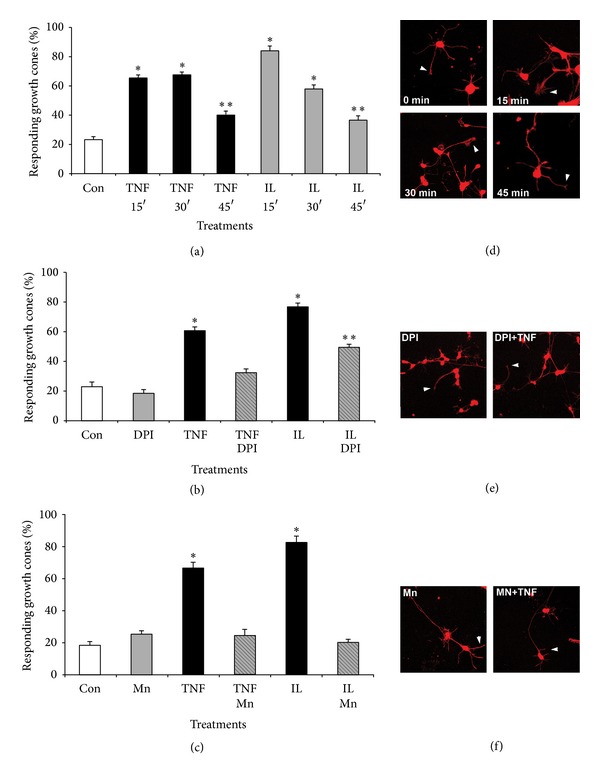
Cytokines elicit redox-dependent reorganization of actin filaments in neuronal growth cones. SC neurons grown on laminin (2 days) were incubated with 40 *μ*M MnTBAP, 5 *μ*M DPI or left untreated prior to addition of TNF*α* or IL-1*β* (200 ng/mL) for increasing time periods (15 min, 30 min, and 45 min). Cultures were fixed, permeabilized (Triton-X-100), and stained with rhodamine phalloidin to reveal filamentous actin. Random images were acquired (40x, confocal microscope), growth cones were scored for the presence of at least one, distinct actin filament-rich structure, and all values normalized to control conditions (% responding growth cones). (a) Both TNF*α* and IL-1*β* significantly increased the percentage of growth cones with actin-filament rich structures (*P < 0.01) at 15 min and 30 min after exposure followed by a significant decrease at 45 min after exposure (**P < 0.01 compared to *t* = 15 min) as opposed to control. (b) Inhibition of NOX activity with 2 *μ*M DPI largely negated the formation of actin-filament rich structures upon exposure to TNF*α* (TNF-DPI) or IL-1*β* (IL-DPI, **P < 0.01) as opposed to TNF*α* only (TNF*α*, *P < 0.01) or IL-1*β* only (IL, *P < 0.01), respectively. Large actin filament-rich structures were not affected by DPI in the absence of cytokines (DPI) compared to control (Con). (c) Scavenging ROS with 10 *μ*M MnTBAP abolished the formation of actin filament-rich structures in response to TNF*α* (TNF*α*-Mn) or IL-1*β* (IL-Mn) when compared to TNF*α* only (TNF, *P < 0.01) or IL-1*β* only (IL, *P < 0.01). The degree of actin filament-rich structures in the presence of MnTBAP alone (Mn) was indistinguishable from control (Con). ∗Significant difference from control and ∗∗significant difference from respective cytokine treatment at *P* < 0.05 by Kruskal Wallis test and Dunnett's *t*-test. (d–f) Cultured SC neurons were treated with pharmacological inhibitors prior to bath application of cytokines, fixed with 4% paraformaldehyde, and stained for actin filaments with rhodamine phalloidin (red signal). (d) Representative images of SC neurons with increasing exposure time to TNF*α*. Actin filament-rich structures in growth cones (lamellipodia) are indicated with arrowheads. (e) Incubation with DPI largely abolished the formation of actin filament-rich structures in SC neurons cultures. (f) Scavenging ROS with MnTBAP effectively negated appearance of actin filament-rich structures in growth cones of SC neurons.

Earlier studies in neuroblastoma cells revealed oxidative damage to actin due to TNF*α* exposure [14]. We found that growth cones responded rapidly to cytokine exposure with changes in morphology prior to collapse suggesting reorganization of actin filaments. We quantified the percentage of growth cones responding to cytokines with at least one distinct actin filament-rich, lamellipodial structure as a function of both time and pharmacological treatment (Figure 9). Growth cones exhibited a rapid, yet transient increase in actin filament-rich structures within 15 min upon exposure to 100 ng/mL TNF*α* (65 ± 5%, *n* = 180, *P < 0.05) or 100 ng/mL IL-1*β* (84 ± 5%, *n* = 180, *P < 0.05) compared to control (23 ± 5%, *n* = 180, ∗*P* < 0.05) and persisted up to 30 min (TNF*α*: 67 ± 5%, *n* = 180, *P < 0.05, and IL-1*β*: 58 ± 5%, *n* = 180, *P < 0.05) (Figures 9(a) and 9(d)). However, the percentage of growth cones with actin filament-rich structures greatly subsided 45 min after addition (TNF*α*: 40 ± 4%, *n* = 180, **P < 0.05, and IL-1*β*: 37 ± 5%, *n* = 180, **P < 0.05) compared to the initial response. Inhibiting NOX activity (5 *μ*M DPI) greatly diminished the percentage of responding growth cones upon bath application of cytokines (DPI + TNF*α*: 32 ± 6%, *n* = 180, and DPI + IL-1*β*: 49 ± 6%, *n* = 180, **P < 0.05) compared to absence of DPI (TNF*α*: 60 ± 6%, *n* = 180, *P < 0.05, and IL-1*β*: 76 ± 6%, *n* = 180, *P < 0.05) measured 15 min after addition of cytokines (Figures 9(b) and 9(e)). The percentage of responding growth cones was not altered by DPI treatment alone (18 ± 6%, *n* = 180) compared to control (23 ± 6%, *n* = 180). Similarly, scavenging ROS with 20 *μ*M MnTBAP also negated the formation of actin filament-rich structures in growth cones exposed to cytokines (Mn + TNF*α*: 24 ± 8%, *n* = 180, and Mn + IL-1*β*: 18 ± 8%, *n* = 180) to levels indistinguishable from control (18 ± 4%, *n* = 180) compared to cytokines alone (TNF*α*: 66 ± 8%, *n* = 180, *P < 0.05, and IL-1*β*: 84 ± 8%, *n* = 180, *P < 0.05) (Figures 9(c) and 9(f)). A presence of MnTBAP did not alter the percentage of growth cones with actin filament-rich structures (18 ± 3%,  *n* = 180). Taken together, these data suggested that TNF*α* and IL-1*β* stimulated a redox-dependent, yet transient reorganization of actin filaments in neuronal growth cones prior to collapse of morphology.

## 4. Discussion

**Figure 10:**
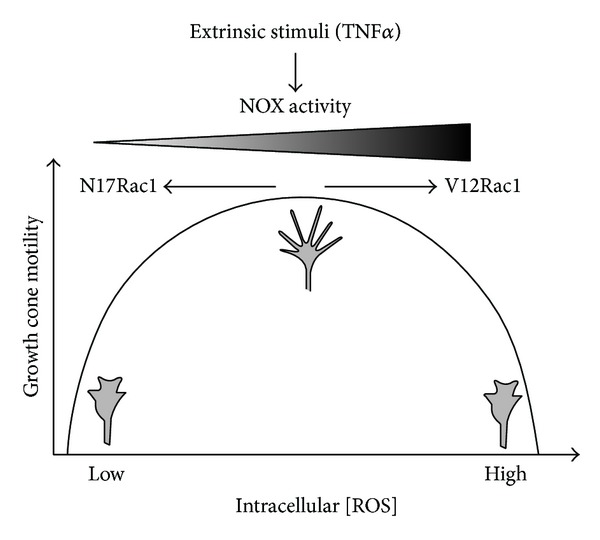
A Rac1-dependent redox rheostat regulates growth cone motility in response to extrinsic stimuli. Exposure of neuronal growth cones either to TNF*α* or to IL-1*β* rapidly stimulates ROS generation through NOX activity under the regulation of the small GTPase Rac1 and concomitant paralysis and degeneration of morphology of growth cones. Moreover, growth cone motility is not only sensitive to the overabundance of ROS either via NOX activation or via constitutive Rac1 activity (Rac1^V12^) but is also impaired by a substantial depletion of ROS (antioxidants) or inhibition of Rac1 activity (Rac1^N17^). Consequently, productive growth cone motility requires an optimal concentration of intracellular ROS generated by a Rac1-regulated NOX activity. It is plausible that this Rac1/NOX-redox rheostat is responsive to many extrinsic stimuli including cytokines, growth factors, hormones, and cell adhesion molecules for which redox signaling mechanism has been demonstrated.

We demonstrated that long-term exposure of SC neurons to the proinflammatory cytokines TNF*α* and IL-1*β* provoked the loss of growth cone motility and the subsequent degeneration of growth cone morphology (collapse). These changes in growth cone advance and behavior translated into the impairment of neurite outgrowth and disruption of process architecture of SC neurons, which was rescued either by scavenging ROS, inhibiting NOX activity, or depleting Rac1 activity despite a presence of TNF*α* or IL-1*β*. Both cytokines stimulated the formation of ROS intermediates and a transient phase of actin filament organization in advancing growth cones. Importantly, ROS intermediates and actin filament reorganization preceded paralysis of growth cone motility and collapse of morphology implying a causative action of this signaling mechanism. Taking into account that exhaustive ROS scavenging (excess of antioxidants) also impaired neurite outgrowth, it is feasible that productive growth cone motility could demand an optimal level of ROS intermediates. We propose that a redox rheostat under the regulation of the Rac1 shapes growth cone motility and hence neurite outgrowth, in response to many extrinsic stimuli other than cytokines (Figure 10).

Prolonged bath application of TNF*α* or IL-1*β* (6–8 h) to dissociated SC neurons or SC explants stunted neurite outgrowth in a dose-dependent manner (Figure 7, Table 1) in accordance with previous findings in hippocampal neurons or neuroblastoma cells [8, 9]. In lieu of the well-documented apoptotic potency of TNF*α* and IL-1*β*, it was imperative to determine whether inhibition of neurite outgrowth had its origin in neuronal cell bodies (apoptosis, necrosis) or directly in the distal compartment of growth cones. Foremost, cytokines were applied to SC neurons after the onset of neurite outgrowth with the majority of processes longer than two cell diameters. Since the number of neurites per neuron (a measure of neurite initiation) remained unaltered and no measureable neuronal cell death occurred over the time period of cytokine exposure (6–8 h) as determined by a live/dead fluorescence assay (calcein green/propidium iodide counterstaining), neither a decrease in neurite initiation nor a decrease in neuronal cell death could account for the observed reduction in neurite outgrowth. Significant neuronal cell death (>15%) was however apparent 24 hours after cytokine addition and increased to over 40% after 2 days. Evidence for direct action of cytokines on growth cone motility and morphology was obtained by live-video phase microscopy (Figures 1 and 2). Acute exposure to TNF*α* or IL-1*β* (bath applied) impaired growth cone motility in a dose-dependent manner within 10 to 15 minutes upon application. Initial paralysis of growth cone advance was followed by degeneration of morphology and subsequent collapse. Restricting cytokine exposure exclusively to advancing growth cones in our bead assay corroborated these findings. In addition, bead assays revealed a transient increase in lamellipodia-like structures or in the vicinity of neuron-bead contacts, which were nonproductive for growth cone advance. This transient increase in actin filaments (Figure 9) was reminiscent of our findings in SH-SY5Y neuroblastoma, yet in the case of primary neurons it revealed a physiological consequence [14]. Direct effects of cytokines on growth cones were further supported by the expression of IL-R1 (IL-1*β* receptor) and TNF-R1 (high affinity p55 TNF*α* receptor) on the entire neuronal surface including cell bodies and growth cones (Figure 1). Together these findings provided strong evidence that TNF*α* and IL-1*β* directly impaired growth cone motility and morphology as the underlying cause for the attenuation of neurite outgrowth over longer time periods of exposure.

Initial evidence for a role of ROS intermediates was suggested by studies demonstrating that antioxidants (ROS scavenging) or DPI (inhibiting NOX-like activities) largely rescued neurite outgrowth, growth cone motility, growth cone morphology, and lastly actin filament organization in the presence of cytokines. Interestingly, neurite outgrowth of SC neurons on laminin exhibited innate redox dependence. Whereas moderate MnTBAP concentrations (5 *μ*M) significantly increased neurite outgrowth, concentration higher than 10 *μ*M stunted neurite outgrowth corroborating studies in Aplysia neurons [15]. It is plausible that moderate MnTBAP concentration (5 *μ*M) scavenges excessive amounts of superoxide derived from mitochondrial respiration and as byproduct of several enzymatic reactions. Under basal culture conditions, this excessive superoxide might compromise optimal growth cone motility (actin dynamics, tubulin dynamics) and hence the observed enhancement of neurite outgrowth by moderate concentrations of scavenger [41–43]. In contrast, higher MnTBAP concentrations could impair vital redox-dependent mechanism necessary for proper neurite outgrowth [44]. Using the oxidation-sensitive fluorescence indicator DCF, we demonstrated substantial yet transient generation of ROS (likely superoxide) in growth cones and cell bodies of SC neurons upon exposure to TNF*α* and IL-1*β* (Figures 3 and 5). Similar results were obtained using the superoxide-specific fluorescence indicator dihydroethidium, yet investigations suffered from considerable neurotoxicity of dihydroethidium (data not shown). Previous studies in chick cortical neurons and human SH-SY5Y neuroblastoma cells demonstrated superoxide production in response to TNF*α* utilizing dihydroethidium oxidation or a SOD-inhibitable cytochrome C oxidation as demonstrated [14, 45]. A significant contribution from nonneuronal cells (microglia) was unlikely since (i) SC neuron cultures contained less than 3% nonneuronal cells, (ii) analyzed cells all exhibited distinct neuronal characteristics (i.e., round cell body, one process longer than 2 cell diameters), and (iii) microglia invade and populate the developing CNS at late embryonic or even postnatal stages [46]. Ratiometric imaging demonstrated significant ROS production in advancing growth cones within 3 to 5 min upon exposure to inflammatory stress as opposed to changes in motility and morphology observed 10 to 15 min following exposure. Several findings strongly suggested a NOX2-like activity as the source of ROS in SC neurons, in particular superoxide [14, 47]. SC neurons exhibited immunoreactivity against NOX2 and the cytosolic subunits p47^phox^ and p67^phox^. A presence of other NOX isoforms can however not be excluded, yet antibody quality against NOX1 or NOX3 was insufficient to produce a conclusive finding. TNF*α* stimulated a dose-dependent increase in ROS intermediates in freshly isolated growth cone particle preparations in conjunction with a presence of NADPH oxidoreductases activity. The phorbol ester PMA as well as TNF*α* induced translocation of the cytosolic subunit p67^phox^ to plasma membranes. Parallel studies on the translocation of the cytosolic subunit p47^phox^ to plasma membranes were hampered by the considerable variability of immunoreactivity. Expression of NOX-like activities was reported in both primary PNS and CNS neurons [48–51]. Lastly, the antioxidant MnTBAP inhibits the TNF*α* or IL-1 *β*-stimulated ROS formation. Also, pharmacological inhibition with DPI was effective in blocking TNF*α* or IL-1*β*-mediated responses of SC neurons including ROS formation and partial rescue of growth motility, neurite outgrowth, growth cone collapse, and actin filament reorganization (Figures 2, 4, 5, 7, and 9). Notably, all these experiments revealed considerable toxicity of DPI and thus, depending on the duration of DPI application, put restrictions on the usable concentrations of DPI. Mechanistically, DPI blocks single electron transport reactions such as NOX-mediated generation of superoxide. Nevertheless, numerous other enzymatic reactions are also compromised foremost electron transport of complex I in mitochondria, which could account for the observed toxicity. Lastly, depleting Rac1 activity negated cytokine-mediated ROS formation in SC neurons, whereas Rac1 overexpression alone was sufficient to increase ROS formation. The small GTPase Rac1 emerged as a pivotal regulator of cytokine-stimulated ROS formation in SC neurons in accordance with previous reports [22, 52, 53]. In our study, depleting Rac1 activity abolished ROS formation in the presence of TNF*α* or IL-1*β* yet without affecting basal ROS levels (Figures 6(b)–6(d)). In contrast, overexpression of constitutively active Rac1 alone was sufficient to stimulated ROS generation in SC neurons and also reduced neurite outgrowth, which was not further exacerbated upon addition of TNF*α* or IL-1*β* (data not shown). Effects of Rac1 depletion or overexpression were addressed by trituration loading of purified recombinant Rac1-GST mutant chimeras since adenoviral-mediated expression of Rac1 mutants required a 3-day expression period incompatible with neurite outgrowth analysis [26, 31]. Not unexpectedly, introduction of Rac1^N17^ or Rac1^V12^ resulted in a measurable reduction in neurite outgrowth on laminin even in the absence of cytokines [11, 26, 54, 55]. In support of our findings, numerous reports link Rac1 as a regulator of ROS intermediates in cellular signal transduction. Neurite extension in PC12 cells upon NGF stimulation encompasses increases in Rac1 activity and H_2_O_2_ formation [56]. A Rac1 mutant lacking residues 124–135 of the insert region blocking ROS generation disrupted membrane ruffling and mitogenesis in fibroblasts and caused a downregulation of RhoA [44, 57–59]. With respect to actin cytoskeleton dynamics, TNF*α* and IL-1*β* induced a transient phase of actin filament reorganization (increases in actin filament density) in neuronal growth cones, which was however nonproductive for motility. Interestingly, actin filament reorganization was not repeatable with another addition of cytokines even after wash-out and extensive recover time suggesting permanent or persistent damage to actin filament reorganization by cytokines. Irreversible oxidative damage (carbonylation) to actin was detectable in neuroblastoma cells exposed to TNF*α* [14]. These findings provided evidence that NOX activation in neuronal growth cones serves as source of ROS intermediates in response to cytokines and that ROS intermediates are likely causative for cytokine-mediated degeneration of neuronal growth cones.

Inflammatory and oxidative stress is a hallmark of neurodegeneration in most chronic, acute, and even some psychiatric CNS pathologies [3, 60, 61]. The proinflammatory cytokines TNF*α* and IL-1*β* are important to orchestrate inflammatory and oxidative stress in the disease and aging CNS although pleiotropic actions in the adult and developing CNS are known, hence establishing a complex and delicate balance between neuroprotection and neurotoxicity. Members of the Nox/Duox family have emerged as [46, 62] key sources of oxidative stress in aging and the progression of many pathologies beyond the CNS and have been recognized as key therapeutic targets [63–66]. Our findings provide support for a role of NOX activity in primary neurons as a principal source of oxidative stress triggered by TNF*α* or IL-1*β* [16]. Overabundance of ROS intermediates is likely to disrupt proper actin filament dynamics, which is vital for the plasticity and morphology of distal neuronal processes including neurite outgrowth, sprouting, and spine dynamics. Strategies to block NOX activities could thus proof beneficial to blunt inflammatory stress in the diseased and aging CNS and to halt cognitive decline [66, 67].

## Figures and Tables

**Table 1:** Redox-sensitive inhibition of neurite outgrowth in response to TNF*α* and IL-1*β*.

Condition	NL_50_ ± SEM	*n*
Control (10 mg/mL Ov)	147 ± 16 *μ*m	104
TNF*α* 40 ng/mL	118 ± 6 *μ*m∗	74
TNF*α* 100 ng/mL	86 ± 13 *μ*m∗	173
TNF*α* 100 ng/mL **+** 10 mM MnTBAP	145 ± 13 *μ*m	62
TNF*α* 100 ng/mL + 2 mM DPI	113 ± 12 *μ*m∗∗	56
Control (10 mg/mL Ov)	128 ± 7 *μ*m	61
IL-1*β* 40 ng/mL	109 ± 4 *μ*m∗	92
IL-1*β* 100 ng/mL	80 ± 3 *μ*m∗	107
IL-1*β* 100 ng/mL + 10 mM MnTBAP	112 ± 9 *μ*m∗∗	113
IL-1*β* 100 ng/mL + 2 mM DPI	87 ± 8 *μ*m∗	78
PBS	128 ± 12 *μ*m	156
MnTABP 5 mM	161 ± 10 *μ*m∗	82
MnTABP 10 mM	133 ± 7 *μ*m	75
MnTABP 20 mM	115 ± 7 *μ*m∗	56
PBS	179 ± 7 *μ*m	103
NAC 2 mM	89 ± 7 *μ*m∗	102

SC neuron cultures were incubated with pharmacological inhibitors (MnTBAP, DPI) or PBS prior to addition of cytokines for 6–8 hours. After fixation, the average neurite length of the longest neurite per neuron reached by 50% of neurons (NL_50_) was quantified for each condition. ∗Significant differences from controls. ∗∗Significant difference from cytokine only at *P* < 0.05 by one-way ANOVA and Dunnett's *t*-test.

## Abbreviations

DCF: 2′,7′-dichlorofluorescein
DMEM: Dulbecco's Modified Eagle Medium
DPI: Diphenylene iodonium
FBS: Fetal bovine serum
GST: Glutathione-S-transferase
IL-1*β*: Interleukin-1 beta
MnTBAP: manganese(III) tetrakis(4-benzoic acid) porphyrin chloride
NAC: N-acetyl-L-cysteine
ROS: Reactive oxygen species
SC: Spinal cord
TBS: Tris-buffered saline
TNF*α*: Tumor necrosis factor alpha.
